# hsa_circ_0081143 promotes cisplatin resistance in gastric cancer by targeting miR-646/CDK6 pathway

**DOI:** 10.1186/s12935-019-0737-x

**Published:** 2019-02-01

**Authors:** Minghui Xue, Guangyan Li, Xiangjie Fang, Lili Wang, Yuhong Jin, Qinglan Zhou

**Affiliations:** 1 Department of Gastrointestinal Surgery, The First Affiliated Hospital of Xinxiang Medical University, NO. 88 Jiankang Road, Weihui, 453100 Henan China; 2 Department of Gastroenterology, The First Affiliated Hospital of Xinxiang Medical University, Weihui, 453100 Henan China; 3 Department of Gastrointestinal Endoscopy, The First Affiliated Hospital of Xinxiang Medical University, Weihui, 453100 Henan China; 4 Department of Orthopedic Surgery, The First Affiliated Hospital of Xinxiang Medical University, Weihui, 453100 Henan China

**Keywords:** hsa_circ_0081143, miR-646, CDK6, Gastric cancer

## Abstract

**Background:**

Increasing studies indicated that circRNAs play critical roles in tumor progression. However, the roles and underlying mechanisms of circRNAs in gastric cancer (GC) remain largely unclear.

**Methods:**

Microarray assay was used to screen the abnormally expressed circRNAs in GC. Cell viability assay, transwell assay and in vivo assay were performed to assess the effects of hsa_circ_0081143 on GC cells. Next, interaction between hsa_circ_0081143 and miR-646 was detected by luciferase reporter assay and RNA pull-down assay.

**Results:**

High throughput microarray assay showed that hsa_circ_0081143 was upregulated in GC tissues, which was further confirmed by qRT-PCR. Correlation analysis showed that high hsa_circ_0081143 expression was associated with the advanced TNM stage, lymphnode metastases, and poor overall survival of GC patients. Hsa_circ_0081143 inhibition decreased GC cells viability, invasion ability and induced the sensitivity of GC cells to cisplatin (DDP) in vitro. Mechanistically, we showed that hsa_circ_0081143 could act as an endogenous sponge by directly binding to miR-646 and downregulation of miR-646 efficiently reversed the inhibition of CDK6 induced by hsa_circ_008114 knockdown. Additionally, hsa_circ_0081143 silencing suppressed the tumorigenesis and remarkably enhance DDP inhibitory effects of GC cells in vivo.

**Conclusions:**

Our study indicated a novel regulatory loop that hsa_circ_0081143/miR-646/CDK6 axis in GC progression. These data suggested that hsa_circ_0081143 might act as a potential novel therapeutic strategy for GC treatment.

## Background

Gastric cancer (GC) is one of the leading causes of cancer-related death worldwide, particularly in China [1, 2]. Currently, surgical resection is still the main option for treating GC [3]. Although therapeutic strategies have been developed and widely used in the past several decades, GC patients’ prognosis still remains unsatisfactory due to metastasis and chemoresistance [4, 5]. Diaminodichloroplatinum (cisplatin, DDP) is one of the most effective and widely used DNA-damaging anticancer drugs used for cancer treatment [6]. Therefore, it is of great significance to identify new diagnostic biomarkers and more effective therapeutic approaches for the treatment of GC.

Circular RNAs (circRNAs) are a type of covalently closed loop structure of endogenous RNAs, which are characterized by linking the 3′ and 5′ ends generated by back splicing [7, 8]. Recently, increasing studies showed that circRNAs could play critical regulatory roles in differentiation, proliferation, invasion and apoptosis [9, 10]. For example, Zong et al. [11] showed that circRNA_102231 was significantly increased and promoted lung cancer cells proliferation and invasion in vitro. Li et al. [12] showed that circFGFR4 promoted differentiation of myoblasts via binding miR-107 to relieve its inhibition of Wnt3a. Jin et al. [13] found that circHIPK3 served as a prognostic marker to promote glioma progression by regulating miR-654/IGF2BP3 signaling. These reports suggested that circRNAs could be valuable diagnostic and therapeutic strategies in GC. Nevertheless, the biological function and underlying mechanisms of circRNAs in GC remain to be further studied.

In the present study, high throughput microarray assay showed that hsa_circ_0081143 was upregulated in GC tissues, which was reported in a previous study [14]. High hsa_circ_0081143 expression was significantly increased and associated with advanced clinical features and poor overall survival of GC patients. Subsequently, we explored the molecular mechanism underlying hsa_circ_0081143 deregulation in GC progression, we identified that hsa_circ_0081143 promoted GC progression via the hsa_circ_0081143-miR-646-CDK6-KLF5 signaling axis, suggesting hsa_circ_0081143 might act as a potential therapeutic target for GC treatment.

## Materials and methods

### Patients and methods

30 paired human GC tissues and adjacent non-tumor tissues were obtained from patients who received surgical treatment at First Affiliated Hospital of Xinxiang Medical University. All tissues were quickly frozen in liquid nitrogen and stored at − 80 C until total RNA extraction. This study was approved by the ethics committee of First Affiliated Hospital of Xinxiang Medical University. Signed written informed consents were obtained from all participants before the study. The clinicopathological features of the GC patients are summarized in Table 1.

**Table 1:** Correlation between hsa_circ_0081143 expression and clinical features of GC patients

Clinicopathological features	Total	hsa_circ_0081143		P
		Low	High	
Age (years)				0.464
< 60	14	6	8	
≥ 60	16	9	7	
Gender				0.273
Male	15	9	6	
Female	15	6	9	
Tumor size (cm)				0.269
< 5	17	10	7	
≥ 5	13	5	8	
Differentiation				0.058
Well	11	8	3	
Moderate + poor	19	7	12	
TNM stage				0.025
I + II	12	9	3	
III + IV	18	6	12	
Lymph node metastasis				0.008
No	19	13	6	
Yes	11	2	9	

### Human circular RNA microarray

After being obtained from surgical specimens, samples (3 pairs of GC tissues and adjacent non-tumor tissues) were immediately frozen using liquid nitrogen. Sample preparation and microarray hybridization were performed according to the protocols of Arraystar (Rockville, MD, USA). The circRNAs chip containing 5396 probes specific for human circular RNAs splicing sites was used. Total RNA was extracted, and digested with Rnase R kit (Epicentre, Madison, WI) to remove linear RNA. Human circRNA microarray hybridization was performed according to Arraystar standard protocols. The enriched circRNA was amplified to cDNA, and transcribed into cRNA using Arraystar Super RNA Labeling Kit (Arraystar, Rockville, MD). Labeled cRNAs were then hybridized using Arraystar Human circRNA Array (8 × 15 K, Arraystar) and scanned by the Agilent Scanner G2505C (Jamul, CA, USA). CircRNAs demonstrating fold-changes of ≥ 2 and P-values of < 0.05 were regarded as significantly differentially expressed.

### Cell culture and transfection

Human GC cell lines SGC7901, MGC803 and Human gastric epithelial cell line (GES1) was purchased from Biochemistry and Cell Biology of the Chinese Academy of Sciences (Shanghai, China). The DDP-resistant SGC7901/DDP cells and MGC803/DDP cells are purchased the KeyGen (Nanjing, China). All cell lines were cultured in Dulbecco’s Modified Eagle Medium (DMEM, Gibco, Carlsbad, CA, USA) supplemented with 10% FBS, 100 U/ml penicillin, and 0.1 mg/ml streptomycin in a 37 °C humidified incubator with 5% CO CO_2_.

The small interfering RNAs (siRNAs) specifically targeting has-circ_0081143 (si- circ_0081143) and scrambled negative control siRNA (si-NC) were obtained from RiboBio (Guangzhou, China). Expression plasmids pcDNA3.0 (Invitrogen) were then constructed and sequenced for the ectopic expression of CDK6 (pcDNA-CDK6). MiR-646 mimics, miR-646 inhibitors and NC mimics and NC inhibitors were designed and synthesized by Gene-Pharma (Shanghai, China). The cells were transfected with RNA oligoribonucleotides or constructs using Lipofectamine 2000 (Invitrogen) according to the manufacturer’s protocol.

### Cell viability assays

Cell proliferation was measured by Cell Counting Kit-8 (CCK-8, Roche, Basel, Switzerland) following the manufacturer’s instructions. Cells (2 × 10^3^/well) were seeded into 96-well plates (Corning, NY, USA) and incubated for 24 h, 48 h, and 72 h, respectively. 10 μL of CCK-8 regents were added into cells and incubated for 2 h at 37 °C. The absorbance was measured at 450 nm on the spectrophotometer.

Colony formation assay was conducted for 14 days, and the colonies were fixed in 70% ethanol for 10 min and then stained with 1% crystal violet solution for another 10 min at room temperature. Images were captured with a camera (Nikon, Japan).

### Flow cytometry

For cell cycle analysis, GC cells (2 × 10^5^ cells) were fixed overnight at 4 °C in 70% ethanol. Then, cells washed with PBS and treated with propidium iodide (PI, 0.05 mg/mL, Sigma, MO, USA) for 20 min at room temperature. Cell cycle analysis was used with FACS flow cytometry (BD Biosciences, NJ, USA).

Cell apoptosis was analyzed with Annexin V-FITC Apoptosis Kit (Biovision, CA, USA) according to the manufacturer’s instructions. After the labeling, Cells were analyzed with FACS flow cytometry (BD Biosciences).

### Transwell invasion assay

The invasion ability was assessed using 6.5-mm transwell chambers with a pore size of 8 μm (Costar, Corning, NY, USA). Cells (2 × 10^5^) were suspended in 100 μL serum-free medium and seeded into the upper chamber or precoated with 80 μL of Matrigel solution (BD, NJ, USA) for cell invasion assay. The lower chamber was filled with 600 μL of 10% FBS medium. After incubation for 48 h, cells that had invaded to the lower side of the membrane were fixed and stained with crystal violet. Five random fields were chosen to count and take photos under a microscope.

### Quantitative real-time PCR (qRT-PCR)

Total RNA was isolated from tissues or cell lines using TRIzol reagent (Invitrogen, Carlsbad, CA, USA) according to the manufacturer’s protocol. Then, the reverse transcription was done with a Reverse Transcription Kit (Takara, Tokyo, Japan). Real-time PCR analysis was performed with SYBR Green (Takara). The amounts of expression level were calculated by the 2^−ΔΔCt^ method and the relative expression level was normalized to the expression of GAPDH (for circRNAs and mRNAs) or U6 (for miRNAs). QRT-PCR reactions were performed by the ABI7500 system (Applied Biosystems, Shanghai, China).

### Western blot analysis

Cells were lysed using RIPA protein extraction reagent (Beyotime, Shanghai, China) supplemented with PMSF (Roche, Basel, Switzerland) and 1% protease inhibitors. The protein concentration is quantified by BCA protein assay kit (Pierce, Rockford, IL, USA). Proteins were separated by 8% sodium dodecyl sulfate–polyacrylamide gel electrophoresis (SDS-PAGE) and transferred to polyvinylidene difluoride (PVDF membranes. The membranes were then incubated with primary antibodies (Cell Signaling Technology, MA, USA) at 4 °C overnight, followed by reaction with corresponding secondary antibody. Chemiluminescence was detected using the ECL kit (Beyotime, China). GAPDH antibody was used as an internal control.

### Immunohistochemical analysis

Formalin-fixed, paraffin-embedded tissue sections were used for the immunohistochemical analysis. Paraffin was removed from the tissues and the sections were hydrated through a graded series of ethanol. Antigen retrieval was and sections were blocked with 5% sheep serum for 60 min. Sections were incubated with antibody (Abcam) overnight at 4 °C. Then signals were visualized with 3,3′-diaminobenzidine on the second day.

### Luciferase reporter assay

A hsa_circ_0081143 segment was synthesized with either mutant or wild type (Wt) seed region and cloned into the psiCHECK-2 vector. Cells (1 × 10^5^ cells/well) were co-transfected with circ_0081143-Wt or circ_0081143-Mut and miR-646 mimics or miR-NC using Lipofectamine 2000 (Invitrogen). After induction for 48 h, luciferase activity was assessed using the dual-luciferase reporter kit (Promega, Madison, WI, USA). A wild-type 3′-UTR fragment of CDK6 (CDK6-Wt) and mutant CDK6 -3′UTR mRNA containing the putative miR-646 binding site was amplified by PCR and cloned into downstream of the firefly luciferase gene in the pMIR-REPORT vector (Thermo Scientific, Waltham, MA, USA). For the luciferase assay, cells were co-transfected in 48-well plates with CDK6-Wt or CDK6-Mut, miR-646 mimics or miR-NC mimics using Lipofectamine 2000 reagents.

### RNA pull-down assay

RNAs were labeled with biotin using Pierce RNA 3′ End Desthiobiotinylation Kit (Thermo Scientific) according to the manufacturer’s protocols. Then biotin-labeled wild-type miR-646 and negative control (mutant miR-646) were incubated with cell lysates, as well as Magnetic beads. After incubation for 6 h, the precipitated RNAs on beads were eluted and extracted, followed by qRT-PCR examination.

### In vivo assay

5-week-old female athymic BALB/c mice were maintained under specific pathogen-free conditions and manipulated according to protocols approved by the animal center of Xinxiang Medical University. They were randomly divided into the following groups: (a) si-NC + PBS; (b) si-circ_0081143 + PBS; (c) si-NC + DDP; (d) si-circ_0081143 + DDP. When the tumors were palpable, DDP (5 mg/kg) was peritoneally injected into the mice every 4 days. Tumor volumes were examined every week when the implantations were starting to grow bigger. All mice were killed 6 weeks after injection.

### Statistical analysis

The statistical analysis was carried out by utilizing SPSS 20.0 statistical software. Data was represented as mean ± the standard deviation (SD). The significance of differences between groups was estimated by two-side student’s t-test, χ^2^ test or ANOVA as appropriate. P < 0.05 was considered as significant.

## Results

### Hsa_circ_0081143 is upregulated in GC

**Fig. 1:**
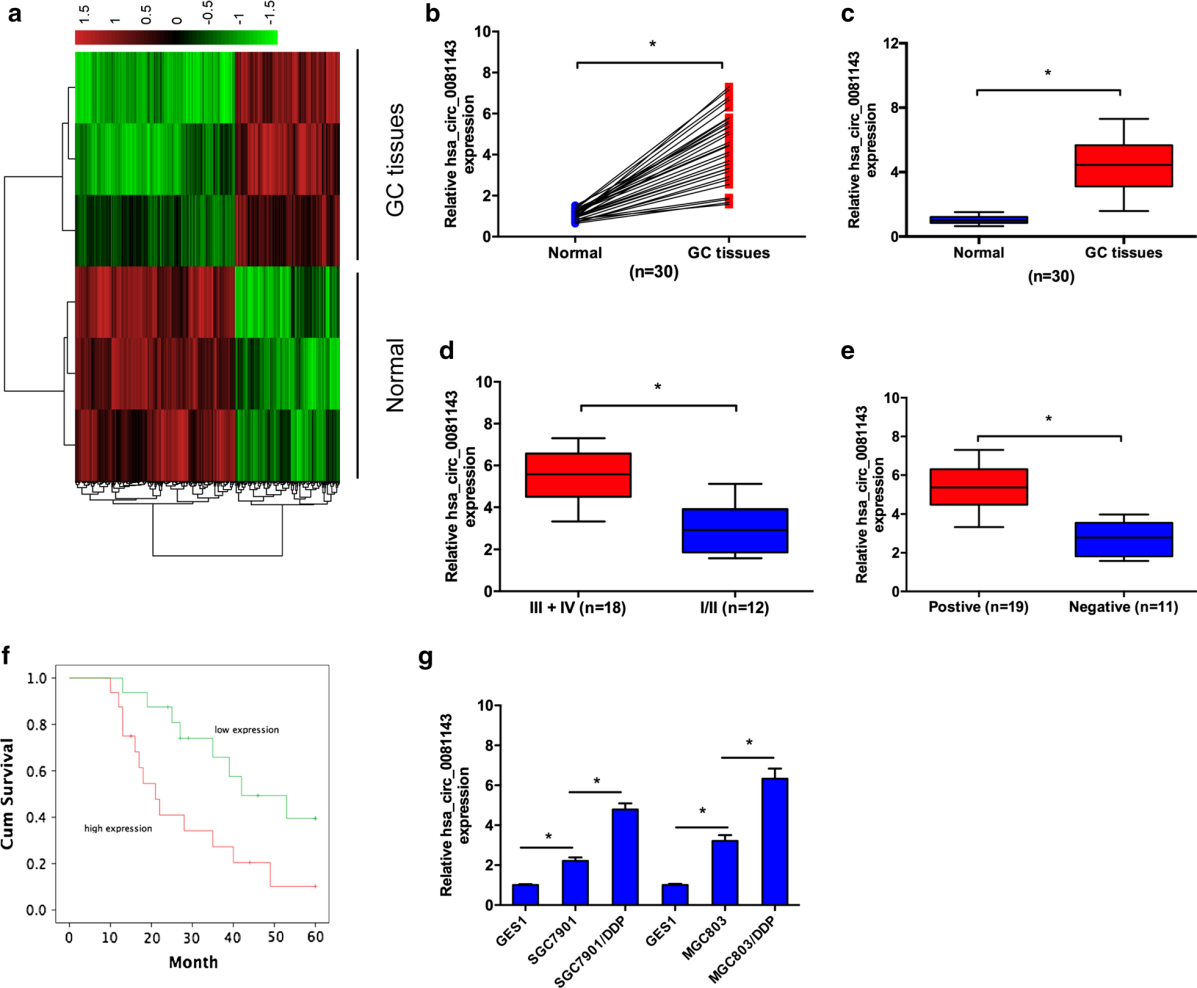
Hsa_circ_0081143 was increased in GC. **a** Heatmap of the most differentially expressed circRNAs in GC tissues compared to normal gastric tissues according to circRNA microarray dataset. **b**, **c** Relative expression of hsa_circ_0081143 in 30 pairs of GC tissues and adjacent non-tumor tissues by qRT-PCR. **d** Relative expression of hsa_circ_0081143 in GC tissues with different TNM stages. **e** Relative expression of hsa_circ_0081143 in GC tissues with lymphnode status. **f** Kaplan–Meier analysis showed that GC patients with high hsa_circ_0081143 expression have a poor prognosis. **g** Relative expression of hsa_circ_0081143 in SGC7901/DDP, MGC803/DDP, SGC7901, MGC803, and GES1 cells. *P < 0.05

Human circular RNA microarray was used to screen the differently regulated circRNAs in 3 pairs of GC tissues. After normalization, 386 circRNAs (151 up-regulated and 235 down-regulated) were aberrantly expressed with fold change ≥ 2.0 and P < 0.05 (Fig. 1a). Hsa_circ_0081143 was one of the most upregulated circRNAs in GC. Next, we explored hsa_circ_0081143 expression in 30 pairs GC tissues. Results showed that hsa_circ_0081143 was significantly increased in GC tissues compared to non-tumor tissues (Fig. 1b, c). Correlation analysis indicated that hsa_circ_0081143 expression was correlated with advanced TNM stage (Fig. 1d) and lymph-node metastasis (Fig. 1e) (Table 1, P < 0.05). Kaplan–Meier analysis indicated that GC patients with high hsa_circ_0081143 expression have a poor overall survival (Fig. 1f). In addition, qRT-PCR showed that hsa_circ_0081143 expression was significantly upregulated in SGC7901/DDP and MGC803/DDP cells compared to SGC7901 and MGC803 cells (Fig. 1g). Collectively, these data suggest that the upregulation of hsa_circ_0081143 may be implicated in GC tumorigenesis.

### Hsa_circ_0081143 promote GC cells progression

**Fig. 2:**
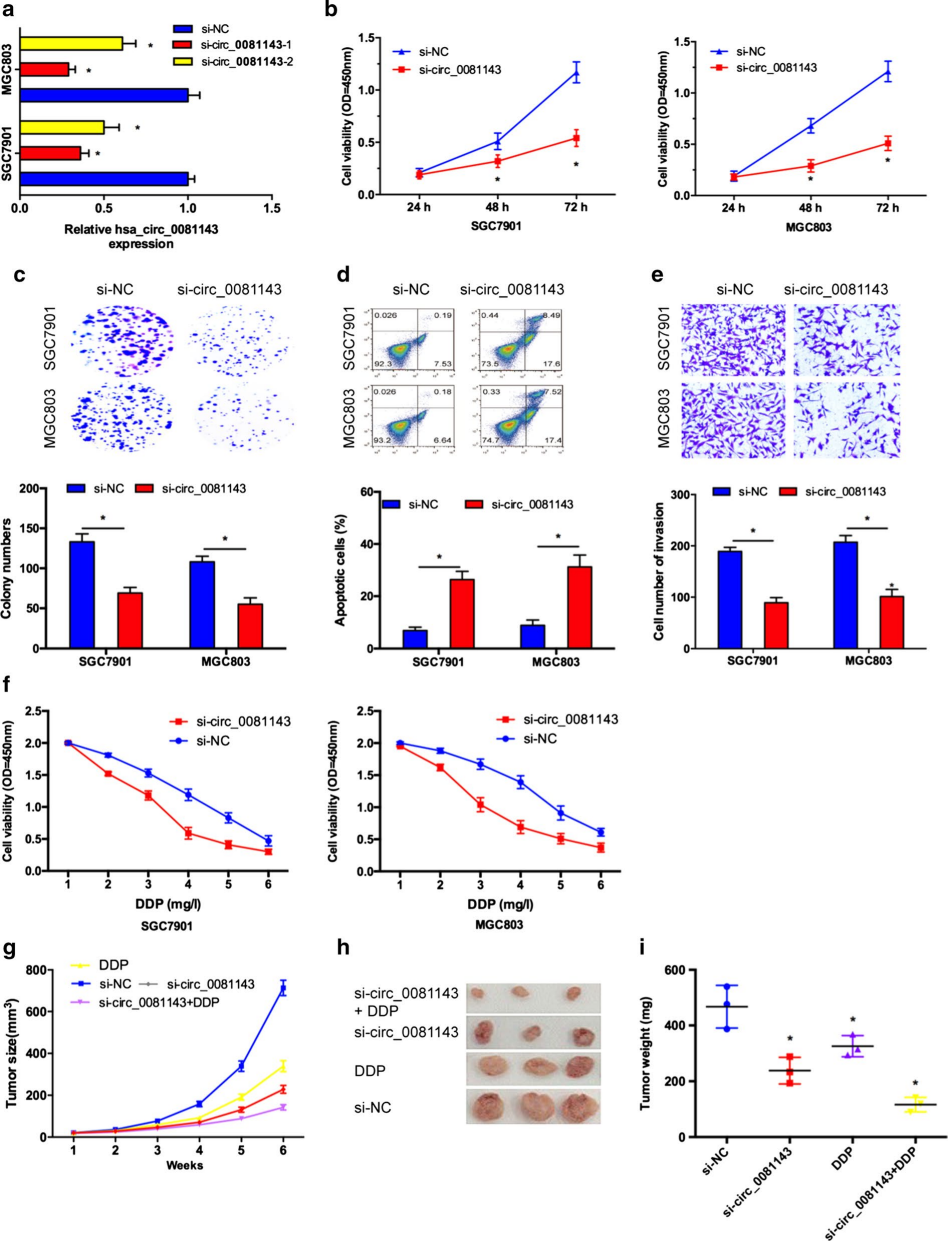
Hsa_circ_0081143 promoted GC cells proliferation, invasion, and cisplatin resistance. **a** Relative expression of hsa_circ_0081143 in GC cells transfected with si-circ_0081143 and si-NC. **b**, **c** Has-circ_0081143 inhibition decreased the proliferation and colony formation ability in GC cells. **d** Has-circ_0081143 inhibition induced GC cells apoptosis rate. **e** Has-circ_0081143 inhibition reduced GC cells invasion ability. **f** Has-circ_0081143 reduction increased the sensitivity of GC cells to cisplatin. **g** The tumor volumes were measured every week. **h** Photographs of tumor xenografts excised 6 weeks. **i** Tumor weight was measured after mouse was surgically dissected. *P < 0.05

To explore the biological effects of hsa_circ_0081143 on GC, we performed loss‐of‐function studies by transfecting with si‐circ_0081143, and chose high-interference siRNA (si‐circ_0081143-1) for subsequent experiments (Fig. 2a). CCK-8 and colony formation assays showed that SGC7901 and MGC803 cells viability in si-circ_0081143 group was significantly reduced compared to si-NC group (Fig. 2b, c). Flow cytometry analysis revealed that hsa_circ_0081143 inhibition increased GC cells apoptosis compared with si-NC group (Fig. 2d). Transwell assay showed that hsa_circ_0081143 inhibition decreased GC cells invasion ability compared with si-NC group (Fig. 2e). In addition, we investigated the effects of hsa_circ_0081143 on the sensitivity of GC cells to cisplatin (DDP). Results showed that si-circ_0081143 significantly induced the sensitivity of SGC7901 and MGC803 cells to cisplatin (Fig. 2f).

Next, we determine the effect of hsa_circ_0081143 on carcinogenesis in vivo, we established xenograft mouse models by subcutaneously injecting MGC803 cells transfected with si-circ_0081143 or si-NC. As shown in Fig. 2g, h, has-circ_0081143 suppression resulted in a smaller size of the subcutaneous tumor in mice, and intraperitoneal injection of cisplatin into the mice with si-circ_0081143 further inhibited the growth of tumor. As expected, the average tumor weight statistic of excised tumor showed a similar trend to tumor volume (Fig. 2i).

### Hsa_circ_0081143 directly contact with miR-646

**Fig. 3:**
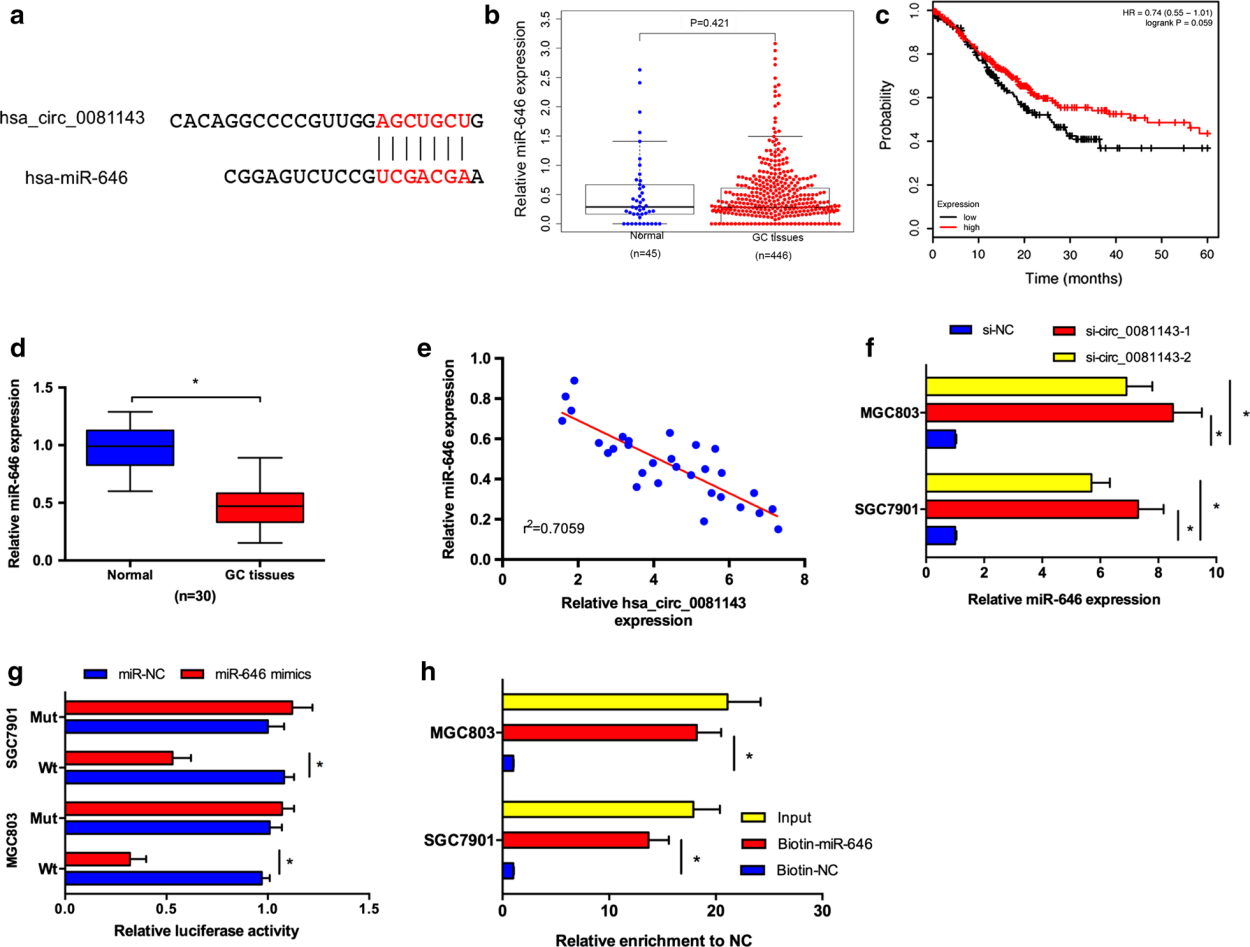
Hsa_circ_0081143 served as a sponge of miR-646. **a** The estimated binding sites of hsa_circ_0081143 and miR-646. **b** TCGA database showed that miR-646 expression was significantly decreased in GC tissues. **c** Kaplan–Meier Plotter revealed that low miR-646 expression was associated with poor overall survival of GC patients. **d** Relative expression of miR-646 in 30 pairs of GC tissues by qRT-PCR. **e** Hsa_circ_0081143 expression was negatively correlated with miR-646 expression in GC tissues. **f** Downregulation of hsa_circ_0081143 suppressed the expression of miR-646 in GC cells. **g** Ectopic expression of miR-646 remarkably reduced the luciferase activity of wild-type but not mutant-type of circ_0081143. **h** RNA pull-down assay revealed that hsa_circ_0081143 was significantly enriched by biotin-miR-646 in GC cells. *P < 0.05

Increasing evidence suggested that some circRNAs are functionally as miRNA sponges to trap miRNA involved in tumorigenesis [15]. To explore whether hsa_circ_0081143 has a similar mechanism in GC, the online software miRBase and circinteractome were used to predict potential miRNAs and miR-646 was selected to examine the relationship between hsa_circ_0081143 and a candidate miRNA (Fig. 3a). TCGA database showed that miR-646 expression was decreased in GC tissues compared to normal tissues. However, the difference is no significant (Fig. 3b). Kaplan–Meier Plotter showed that low miR-646 expression was correlated with poor overall survival of GC patients (Fig. 3c). Next, we explored miR-646 expression in GC tissues, qRT-PCR results revealed that miR-646 expression was significantly downregulated and negatively correlated with hsa_circ_0081143 levels in GC tissues (Fig. 3d, e). Hsa_circ_0081143 inhibition significantly increased miR-646 expression in SGC7901 and MGC803 cells (Fig. 3f). Luciferase reporter assay revealed that miR-646 mimics significantly decreased the activity of circ_0081143-Wt in GC cells (Fig. 3g). Moreover, pull-down assay revealed that hsa_circ_0081143 expression was significantly enriched by biotin-miR-646 in GC cells (Fig. 3h). Taken together, these data indicated that hsa_circ_0081143 might act as a sponge for miR-646 in GC progression.

### CDK6 is target of miR-646 in GC

**Fig. 4:**
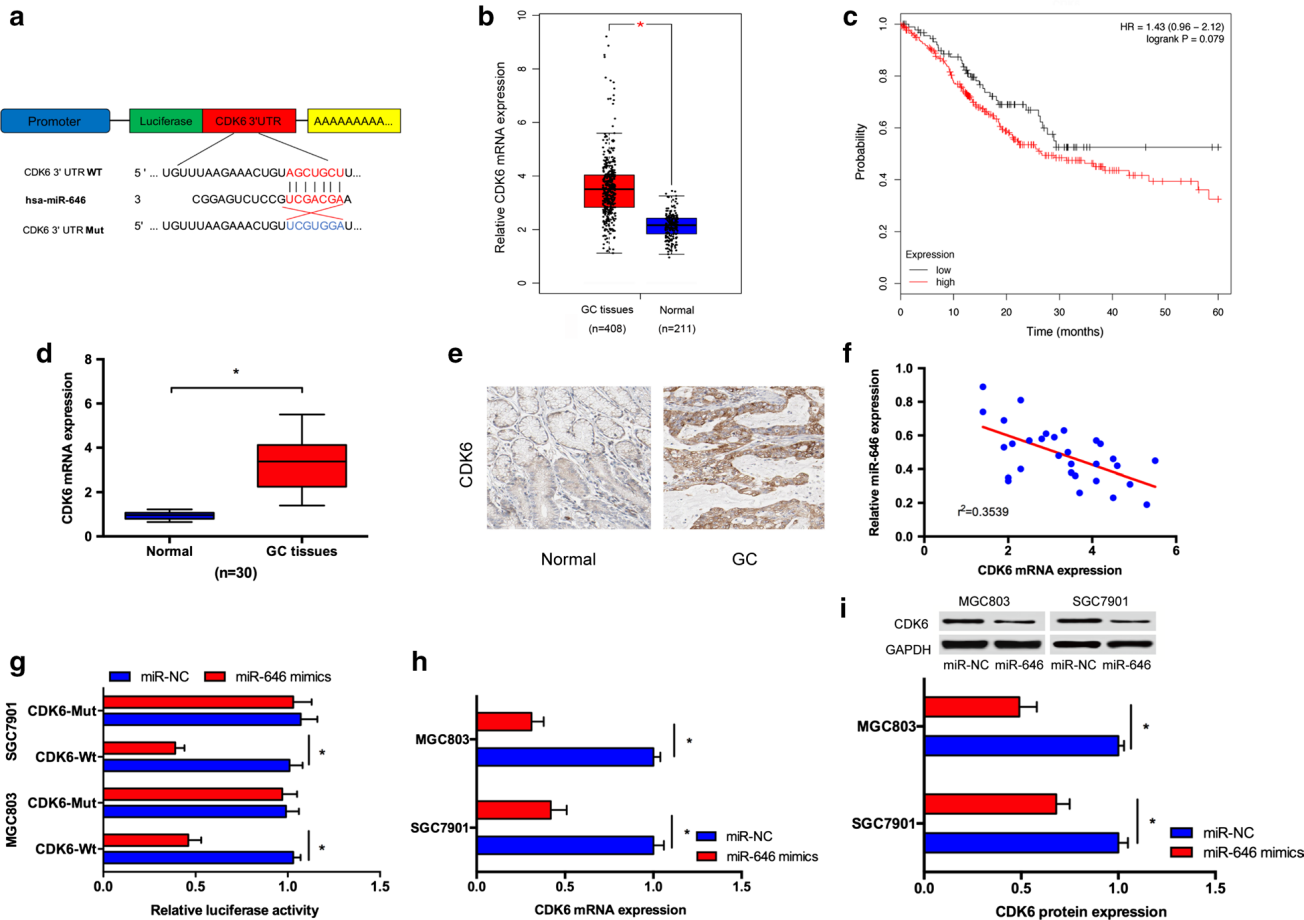
CDK6 is a direct target for miR-646. **a** The estimated binding sites of CDK6 and miR-646. **b** TCGA database showed that CDK6 expression was significantly upregulated in GC tissues. **c** Kaplan–Meier Plotter revealed that high CDK6 expression was associated with poor overall survival of GC patients. **d** QRT-PCR showed CDK6 expression was significantly decreased in GC tissues. **e** IHC results showed that CDK6 protein expression was increased in GC tissues. **f** An inverse correlation was found between the expression level of miR-646 and CDK6. **g** MiR-646 mimics decreased the luciferase activity of wild-type 3′-UTR of CDK6. **h**, **i** Relative CDK6 mRNA and protein levels in GC cells treated with miR-646 mimics were determined by qRT-PCR and western blotting, respectively. *P < 0.05

Subsequently, by using a computational screen for genes with complementary sites of miR-646 in their 3′-UTR using online softwares including miRanda and TargetScan, cell division protein kinase 6 (CDK6) was predicted as a putative target of miR-646 (Fig. 4a). TCGA database showed that CDK6 expression was significantly increased in GC tissues compared to normal tissues (Fig. 4b). Kaplan–Meier Plotter revealed that high CDK6 expression was associated with poor overall survival of GC patients (Fig. 4c). Furthermore, we showed that CDK6 expression was significantly upregulated in GC tissues compared to non-tumor tissues both in mRNA and protein levels (Fig. 4d, e). Spearman’s correlation analysis suggested that miR-646 and CDK6 expression had an inverse relationship in GC tissues (Fig. 4f). Luciferase report assay showed that miR-646 mimics remarkably reduced the luciferase activity of CDK6-Wt reporter (Fig. 4g). Furthermore, we disclosed that miR-646 mimics could trigger a significant inhibitory effects on CDK6 mRNA and protein levels in GC cells (Fig. 4h, i).

### Hsa_circ_0081143 regulates CDK6 expression by competing for miR-646

**Fig. 5:**
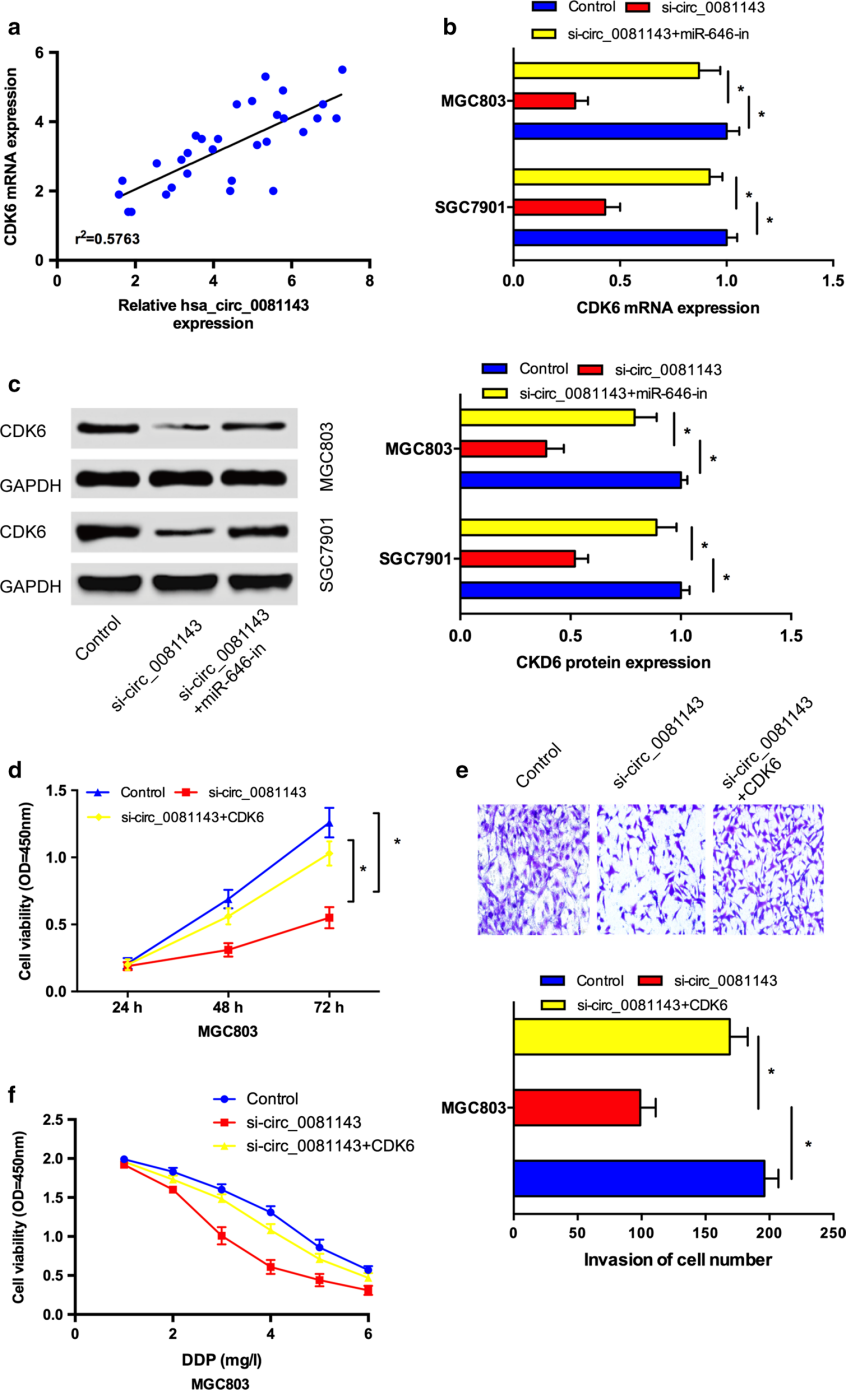
Hsa_circ_0081143 regulated GC progression by modulating miR-646/CDK6 axis. **a** Hsa_circ_0081143 expression was positively correlated with CDK6 expression in GC tissues. **b**, **c** Hsa_circ_0081143 suppression decreased CDK6 expression both in mRNA (**b**) and protein (**c**) levels, while miR-646 inhibitors reversed it. **d** CCK-8 assay showed that hsa_circ_0081143 inhibition reduced GC cells proliferation which could be reversed by CDK6 overexpression. **e** Transwell assay showed that hsa_circ_0081143 inhibition reduced GC cells invasion ability which could be abolished by CDK6 upregulation. **f** The sensitivity of si-circ_004451 transfected GC cells to cisplatin could be reversed by CDK6 overexpression. *P < 0.05

**Fig. 6:**
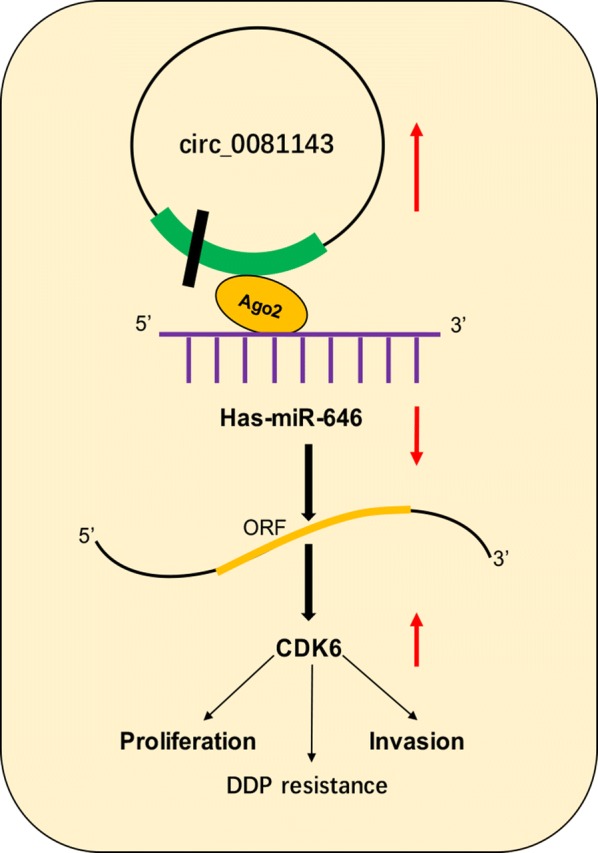
Schematic representation of the proposed mechanism of hsa_circ_0081143 in GC

Studies showed that hsa_circ_0081143 suppressed miR-646 expression and miR-646 targeted CDK6 in GC cells. Then, we explore whether hsa_circ_0081143 regulates CDK6 expression via targeting miR-646. Correlation analysis showed that hsa_circ_0081143 expression was positively correlated with CDK6 expression in GC tissues (Fig. 5a). Moreover, we showed that CDK6 expression both in mRNA and protein levels were markedly restrained after transfecting with si-circ_0081143, and the inhibitory effects of si-circ_0081143 could be obviously reversed by co-transfection with miR-646 inhibitors (miR-646-in) in GC cells (Fig. 5b, c). In addition, rescue experiments were performed. CCK-8 and transwell invasion assays showed that si-circ_0081143 obviously impaired cell proliferation and invasion of MGC803 cells. Upregulation of CDK6 by plasmid restored the effects induced by has-circ_008114 depletion (Fig. 5d, e). Moreover, the sensitivity of si-circ_004451 transfected MGC803 cells to cisplatin could be reversed by CDK6 overexpression (Fig. 5f). Taken together, these data indicated that hsa_circ_0081143/miR-646/CDK6 signaling pathway play important roles in GC progression (Fig. 6).

## Discussion

Recently, increasing evidence indicated that circRNAs play critical roles in GC progression. For example, Chen et al. [16] showed that hsa_circ_0000190 expression was downregulated and associated with advanced tumor progression and poor overall survival of GC patients. Sun et al. [17] showed that circPVRL3 suppression promoted the proliferation and migration of GC cells. Zhang et al. [18] showed that circular RNA_LARP4 inhibited GC cells proliferation and invasion by regulating miR-424-5p/LATS1 axis. However, the expression and underlying mechanism of circRNAs in GC tumorigenesis remain largely unclear.

In the present study, we showed that has-circ_0081143 expression was remarkably increased and correlated with advanced TNM stage, lymphnode metastasis, and poor overall survival in GC patients. QRT-PCR showed that hsa_circ_0081143 expression was significantly increased in SGC7901/DDP and MGC803/DDP cells. Furthermore, we explored the roles of hsa_circ_0081143 on GC progression. Results showed that hsa_circ_0081143 promoted GC cells proliferation, invasion, and reduced the sensitivity of GC cells to cisplatin in vitro. In addition, in vivo assays revealed that hsa_circ_0081143 suppression reduced GC cells growth and enhanced sensitivity of GC cells to cisplatin. These data indicated that hsa_circ_0081143 might exhibit an oncogene-like function in the modulation of GC progression.

As revealed by more and more evidences, circRNAs could act as competing endogenous RNAs (ceRNA) to compete for miRNAs, thereby negatively modulating miRNA expression [19]. Through online software miRBase and circinteractome, miR-646 was identified as a potential target of hsa_circ_0081143. Hsa_circ_0081143 inhibition significantly increased miR-646 expression in GC cells. Luciferase reporter assay revealed that miR-646 mimics significantly decreased the activity of circ_0081143-Wt in GC cells. Pull-down assay further showed that hsa_circ_0081143 expression was significantly enriched by biotin-miR-646 in GC cells. Previous studies showed that miR-646 exhibited a tumor suppressor role in human tumors. For example, Sun et al. [20] showed that miR-646 suppressed osteosarcoma cell metastasis by downregulating fibroblast growth factor 2 (FGF2). Pan et al. [21] showed that miR-646 decreased cell proliferation and metastasis by EGFR pathway in lung cancer. Recently, Zhang et al. [22] revealed that miR-646 inhibited GC cells proliferation and EMT progression by targeting FOXK1. In the present study, our data showed that miR-646 expression was significantly decreased and negatively correlated with hsa_circ_0081143 levels in GC tissues. Kaplan–Meier Plotter revealed that low miR-646 expression was correlated with poor overall survival of GC patients. These data suggested that hsa_circ_0081143 could act as an endogenous sponge to modulate the expression and function of miR-646 in GC progression.

Cell division protein kinase 6 (CDK6), a member of the CDK family, which play critical roles in cell cycle progression, apoptosis, cancer invasion, metastasis, and chemoresistance [23, 24]. For example, Lu et al. [25] showed that miRNA-186 inhibited prostate cancer cell proliferation and tumor growth by targeting YY1 and CDK6. Zhu et al. [26] found that miR-145 sensitized ovarian cancer cells to paclitaxel by targeting Sp1 and CDK6. Moreover, Deng et al. [27] found that miR-218 suppressed GC cells cycle progression through CDK6/Cyclin D1/E2F1 axis. However, the roles of CDK6 expression in GC progression remain unclear. In our mechanistic research, CDK6 was identified as a direct target for miR-646 in GC. Notably, recently some studies pointed out that circRNAs are also implicated in the modulation of CDK6 in human malignances [28–30]. We therefore examined whether hsa_circ_0081143 could affect the expression of CDK6 in GC. As expected, in this study, a positive association between hsa_circ_0081143 and CDK6 was found in GC specimens. CDK6 was found to be positively regulated by hsa_circ_0081143, indicating that CDK6 could be a downstream effector in the hsa_circ_0081143-miR-646 axis. Moreover, rescue experiments further revealed the antagonistic roles of si_circ_0081143 and CDK6 in the modulation of carcinogenic properties of GC cells. Taken together, these data indicated that hsa_circ_0081143/miR-646/CDK6 signaling pathway play important roles in GC progression.

## Conclusion

In the present study, we verified that the knockdown of hsa_circ_0081143 reduced the proliferation, invasion, and cisplatin resistance in GC cells. More importantly, we suggested that hsa_circ_0081143 might act as a ceRNA to inhibit the expression and activity of miR-646, resulting in elevated expression of CDK6. Overall, our findings revealed that hsa_circ_0081143 might serve as an effective therapeutic candidate for GC treatment.
